# A VELOUR post hoc subset analysis: prognostic groups and treatment outcomes in patients with metastatic colorectal cancer treated with aflibercept and FOLFIRI

**DOI:** 10.1186/1471-2407-14-605

**Published:** 2014-08-20

**Authors:** Ian Chau, Florence Joulain, Sheikh Usman Iqbal, John Bridgewater

**Affiliations:** Department of Medicine, Royal Marsden Hospital, London and Surrey, UK; Sanofi, Research and Development, Chilly Mazarin, France; Sanofi, Cambridge, MA USA; Department of Oncology, University College London Hospitals, London, UK

**Keywords:** Aflibercept, Metastatic colorectal cancer, Chemotherapy, Anti-VEGF agents, Targeted therapy

## Abstract

**Background:**

The VELOUR study demonstrated a survival benefit for FOLFIRI + aflibercept versus FOLFIRI + placebo in metastatic colorectal cancer (mCRC) patients who progressed on oxaliplatin-based chemotherapy. Continued divergence of overall survival (OS) curves in the intension to treat (ITT) population, with the survival advantage persisting beyond median survival time, suggested subpopulations might have different magnitudes of survival gain. Additionally, 10% of patients within VELOUR had recurrence during or within 6 months of completing oxaliplatin-based adjuvant therapy (adjuvant fast relapsers) - previously identified as having poorer survival outcomes.

**Methods:**

To determine which patients received the greatest benefit from FOLFIRI-aflibercept, a post hoc multivariate analysis of the VELOUR ITT population was conducted. Prognostic factors identified were applied to the ITT population, excluding adjuvant fast relapsers, to derive OS prognostic profiles.

**Results:**

The better efficacy subgroup was identified as patients within VELOUR exclusive of adjuvant fast relapsers and had performance status (PS) 0 with any number of metastatic site or PS 1 with <2 metastatic site. A significant improvement in efficacy outcome was observed with aflibercept in the better efficacy subgroup. Median OS for FOLFIRI-aflibercept and FOLFIRI-placebo:16.2 and 13.1 months (adjusted Hazard Ratio [HR] = 0.73; 95% confidence interval [CI]: 0.61–0.86); median progression free survival (PFS): 7.2 and 4.8 months (adjusted HR = 0.68; 95% CI: 0.57-0.80); and objective response rate (ORR): 24% versus 11% respectively. Poorer efficacy subgroup comprised of adjuvant fast relapsers or patients with PS2 or PS1 with ≥2 metastatic sites. In poorer efficacy subgroup, no benefit was seen with aflibercept. Median OS for FOLFIRI-aflibercept and FOLFIRI-placebo: 10.4 and 9.6 months (adjusted HR = 0.97; 95% CI: 0.78-1.21) respectively with no improvement in PFS or ORR.

**Conclusion:**

This analysis suggests that within VELOUR, patients in the better efficacy subgroup may derive enhanced benefit from treatment with FOLFIRI-aflibercept. These prognostic criteria may guide practitioners toward optimal use of targeted biologicals in appropriate second-line mCRC patients.

**Electronic supplementary material:**

The online version of this article (doi:10.1186/1471-2407-14-605) contains supplementary material, which is available to authorized users.

## Background

Colorectal cancer (CRC) accounts for more than 1.2 million new cancer cases and over 600,000 deaths [1]. Among these cases, approximately 25% of patients present with metastatic CRC (mCRC) at diagnosis and up to half of all patients with CRC will develop metastases during the course of their disease [2]. Metastatic unresectable CRC is generally incurable and has a 5-year survival rate of only 10-15% [3, 4]. In most patients the disease will progress on initial treatment, and up to half of all patients with mCRC will receive second- and third-line treatments [2, 5]. Better second-line treatments for mCRC are currently needed in this crucial area of high unmet medical need.

A number of treatment options exist for patients whose disease progresses after initial therapy [2, 6, 7], and current real-world treatment patterns show that targeted agents plus standard second-line chemotherapy are commonly utilised [5]. Aflibercept (known as ziv-aflibercept in the United States) is a novel fusion protein containing vascular endothelial growth factor (VEGF)–binding portions from the extracellular domains of human VEGF receptors 1 and 2, fused to the Fc portion of human immunoglobulin (Ig) G1. Aflibercept blocks the activity of VEGFA, VEGFB, and placental growth factor (PlGF) by acting as a high-affinity ligand trap, preventing these ligands from binding to their endogenous receptors [8]. Aflibercept is a new option in the second-line treatment of mCRC and compared to placebo, it has demonstrated statistically significant improvements in overall survival (OS) (median 13.50 versus 12.06 months; hazard ratio [HR] = 0.817, p = 0.0032), progression-free survival (PFS) (6.90 versus 4.67 months; HR = 0.758, p < 0.0001), and overall response rate (ORR) [19.8% (95% confidence interval [CI]: 16.4-23.2) versus 11.1% (95% CI: 8.5-13.8); p < 0.0001] when used in combination with FOLFIRI (5-fluorouracil-leuocovorin-irinotecan) in patients with mCRC that is resistant to or has progressed after an oxaliplatin-containing regimen [9]. The pre-specified subgroup analyses in the VELOUR trial showed a consistent benefit of aflibercept across all major subgroups, including patients who had previously received bevacizumab treatment [10]. Continued divergence of OS curves in the intention to treat (ITT) population, with the survival advantage persisting beyond median survival time, suggested subpopulations might have different magnitudes of survival gain.

The identification of patients who are likely to derive more benefit from targeted anti-angiogenic therapies has become increasingly crucial. Despite more than a decade of intense research effort, no tissue-based predictive biomarkers have yet been established for anti-angiogenic therapy. An optimised treatment approach involving careful patient selection based on identified prognostic factors and known tumor characteristics may enable a more tailored approach to individual treatment. A growing body of evidence indicates that patient characteristics, such as age, performance status, time to recurrence following adjuvant therapy, extent of metastases, and use of prior therapies, can all have an impact on treatment efficacy in mCRC [2]. With these advances in the knowledge of prognostic factors in mCRC, there exists an opportunity in the real-world setting to target patient populations based on clinically relevant phenotypes and prognostic variables. The objective of this post hoc subgroup analysis described herein was to identify a “better efficacy” patient population within VELOUR study that may have derived greater clinical benefit from treatment with FOLFIRI plus aflibercept.

## Methods

This was a post hoc analysis of data from the VELOUR clinical trial which assessed 1,226 patients with histologically or cytologically proven colorectal adenocarcinoma and who had previously received and progressed following oxaliplatin-based chemotherapy [9]. A pre-specified multivariate analysis was conducted on the ITT population in the VELOUR trial to identify prognostic factors associated with improved OS. Trial registration for VELOUR was NCT00561470 and registration date was 20 November 2007.

### Outcome measures

The primary end point of this analysis was OS in the better and poorer efficacy subgroup of patients identified in the multivariate analysis. OS was defined as the time interval from randomisation to death from any cause. PFS was defined as the time interval from randomisation to tumor progression or death from any cause, whichever comes first. Response was assessed according to Response Evaluation Criteria In Solid Tumors (RECIST) version 1.0 [11] based on independent radiology review, blinded to patient treatment.

### Statistical analyses

Efficacy analyses were conducted in the randomised population according to the arm to which patients were assigned at randomisation; response rate was assessed in the subset of these patients who had measurable disease at baseline. Safety was analysed using descriptive methods, in the treated population and subgroups, according to the treatment received (patients who received ≥1 dose of aflibercept were analyzed in the aflibercept arm, regardless of treatment assignment). Time-to-event parameters were estimated using Kaplan-Meier analysis [12]. The hazard ratio (HR) and 95% confidence interval (CI) estimates were provided using a Cox proportional hazards model [13]. Covariates used were based on pre-specified subgroups, including: Eastern Cooperative Oncology Group [ECOG] performance status (PS), prior bevacizumab status, age, prior hypertension, and number of metastatic organs involved. Formal statistical interaction testing was performed between treatment and different prognostic factors at the 2-sided 10% level.

## Results

### Identification of the better efficacy subgroup

The multivariate analysis identified PS and number of metastatic sites as key prognostic factors influencing OS in the VELOUR trial (Table 1). Based on the standardised literature review, the subgroup of patients who relapsed at or within 6 months of adjuvant oxaliplatin-based chemotherapy (adjuvant fast relapsers; n = 124 patients; ~10% of ITT population) were excluded in view of their poor prognosis [14]. At the time of regulatory approval by European Medicine Agency, a survival analysis excluding adjuvant fast relapsers was specifically requested by the Committee for Medicinal Products for Human Use and these results are included in the Summary of Product Characteristics.

**Table 1:** **Results from the pre**-**specified multivariate Cox model**

Prognostic factors	Hazard ratio (95.34% CI)	p-value
Treatment: FOLFIRI-aflibercept versus FOLFIRI-placebo	0.817 [0.713; 0.936]	0.0032
ECOG PS: 1 vs 0	1.683 [1.465; 1.935]	< 0.0001
ECOG PS: 2 vs 0	3.625 [2.395; 5.486]	<0.0001
Prior bevacizumab: yes vs no	1.165 [1.003; 1.353]	0.0425
Age (years): ≥65 vs <65	1.179 [1.020; 1.363]	0.0233
Prior hypertension: yes vs no	0.785 [0.682; 0.904]	0.0007
Number of metastatic organs involved as per IRC: >1 vs (0–1)	1.446 [1.258; 1.663]	< 0.0001

*ECOG*, European Cooperative Oncology Group; FOLFIRI, 5-fluorouracil–leucovorin–irinotecan; *IRC*, independent review committee; *PS*, performance status.

**Figure 1:**
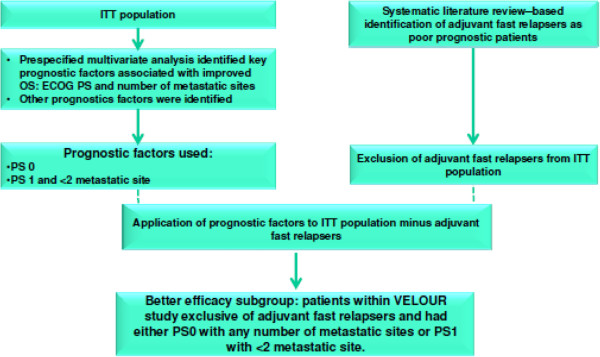
**Construction of the prognostic profile for the better efficacy subgroup.** ECOG: European Cooperative Oncology Group; OS: overall survival; PS: Performance Status; ITT: Intention to Treat.

Prognostic profiles associated with OS, i.e. PS and number of metastases (identified from the pre-specified multivariate analysis), were applied to the ITT population after exclusion of adjuvant fast relapsers. To test the robustness of this prognostic model, the survival of adjuvant fast relapsers and the non-adjuvant fast relapsers within VELOUR was examined according to treatment arm. In non-adjuvant fast relapsers, the HR was 0.78 (95% CI: 0.68-0.90) in favour of aflibercept whereas for adjuvant fast relapsers, HR was 1.12 (95% CI: 0.72-1.74) with no survival benefit seen with aflibercept. A qualitative interaction (although not statistically significant) was noted between treatment and adjuvant fast relapsers likely due to the relative small number (~10%) of adjuvant fast relapsers within the VELOUR ITT population.In the ITT excluding adjuvant fast relapsers population, the interaction between the number of organs with metastases and treatment was significant at the 10% level in the PS1 subgroup (p = 0.0725), whereas no interaction was found in the PS0 subgroup (p = 0.5151), indicating that the treatment effect is different according to the number of metastatic sites in patients with PS1 but not in patients with PS0. Based on the strength of association of the covariates, patients with a performance status of PS0 or PS1 with <2 metastatic site were defined as better efficacy subgroup. Figure 1 shows the construction of the prognostic profile for the better efficacy subgroup. Therefore the final better efficacy subgroup was comprised of patients within VELOUR study exclusive of adjuvant fast relapsers and had either PS0 with any number of metastatic sites or PS1 with <2 metastatic site. The interaction between treatment and the better efficacy subgroup was statistically significant (p = 0.0147) indicating that there was a differential OS effect of aflibercept in this better efficacy subgroup compared to placebo. The poorer efficacy subgroup comprised of patients within VELOUR study who were adjuvant fast relapsers or PS 1 with ≥2 metastatic sites or PS2 with any number of metastatic sites.

The better efficacy subgroup consisted of 406 patients (66%) from the FOLFIRI-placebo control arm and 404 patients (66%) from the FOLFIRI-aflibercept investigational arm of the VELOUR trial. Table 2 shows baseline characteristics, which were similar in both study arms. Within the better efficacy subgroup, 67% of patients received no prior treatment with bevacizumab.

**Table 2:** **Baseline characteristics of patients in the post hoc subgroup analysis**

Parameter	Better efficacy subgroup		ITT population	
	FOLFIRI-Placebo	FOLFIRI-Aflibercept	FOLFIRI-Placebo	FOLFIRI-Aflibercept
	( ***n*** = 406)	( ***n*** = 404)	( ***n*** = 614)	( ***n*** = 612)
Gender, *n* (%)				
Female	174 (42.9)	153 (37.9)	261 (42.5)	247 (40.4)
Male	232 (57.1)	251 (62.1)	353 (57.5)	365 (59.6)
Mean age, y (range)	59.9 (25–86)	59.4 (21–82)	60.2 (19–86)	59.5 (21–82)
Age group, *n* (%)				
<65 y	254 (62.6)	277 (68.6)	376 (61.2)	407 (66.5)
≥65 but <75 y	133 (32.8)	108 (26.7)	199 (32.4)	172 (28.1)
≥75 y	19 (4.7)	19 (4.7)	39 (6.4)	33 (5.4)
Race, *n* (%)				
Asian	29 (7.1)	23 (5.7)	51 (8.3)	35 (5.7)
Black	14 (3.4)	11 (2.7)	27 (4.4)	16 (2.6)
White	355 (87.4)	361 (89.4)	523 (85.2)	548 (89.5)
Other	8 (2)	9 (2.2)	13 (2.1)	13 (2.1)
Cancer diagnosis category, *n* (%)				
Colon	187 (46.1)	188 (46.5)	302 (49.2)	289 (47.2)
Rectosigmoid	95 (23.4)	85 (21)	136 (22.1)	123 (20.1)
Rectum	123 (30.3)	129 (31.9)	174 (28.3)	197 (32.2)
Other	1 (0.2)	2 (0.5)	2 (0.3)	3 (0.5)
Number of organs with metastasis, *n* (%)				
0	2 (0.5)	2 (0.5)	6 (1.0)	2 (0.3)
1	237 (58.4)	230 (56.9)	271 (44.1)	256 (41.8)
>1	167 (41.1)	172 (42.6)	337 (54.9)	354 (57.8)
Metastatic sites, *n* (%)				
Liver	283 (69.7)	294 (72.8)	431 (70.2)	459 (75.0)
Lung	160 (39.4)	152 (37.6)	277 (45.1)	271 (44.3)
Lymph	88 (21.7)	100 (24.8)	181 (29.5)	173 (28.3)
Liver metastasis only, *n* (%)	134 (33)	141 (34.9)	146 (23.8)	153 (25.0)
ECOG performance status, *n* (%)				
0	314 (77.3)	317 (78.5)	350 (57.0)	349 (57.0)
1	92 (22.7)	87 (21.5)	250 (40.7)	250 (40.8)
2	0	0	14 (2.3)	13 (2.1)
Prior bevacizumab, *n* (%)				
Yes	133 (32.8)	132 (32.7)	187 (30.5)	186 (30.4)
No	273 (67.2)	272 (67.3)	427 (69.5)	426 (69.6)

*ECOG*, European Cooperative Oncology Group; FOLFIRI, 5-fluorouracil–leucovorin–irinotecan; *ITT*, intent to treat; PS, performance status.

### Efficacy

**Figure 2:**
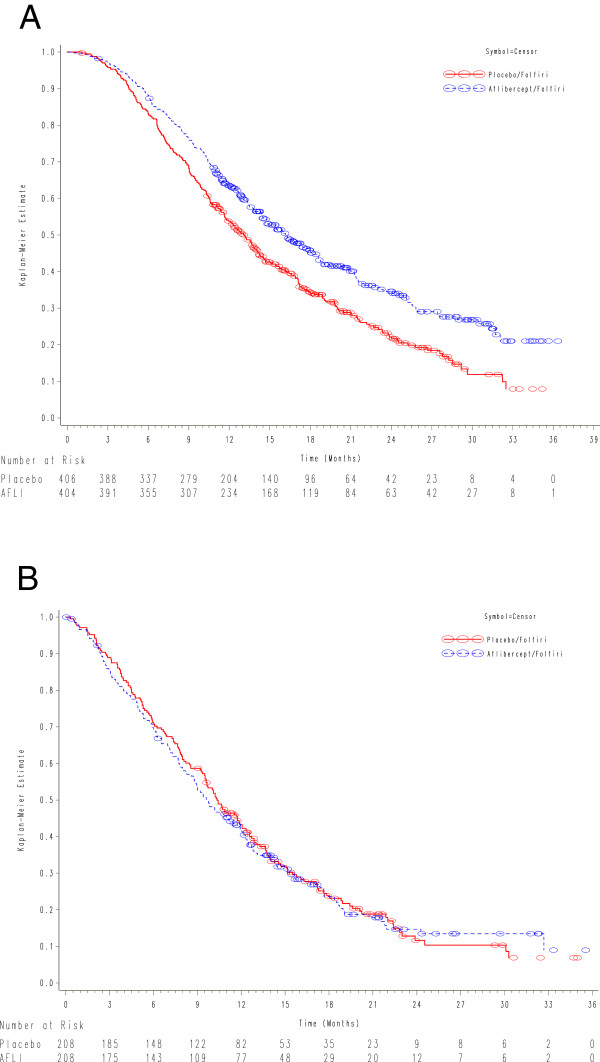
**Overall survival according to treatment arms.** **(A)** Better efficacy subgroup **(B)** Poorer efficacy subgroup.

Figure 2A shows OS for the better efficacy subgroup by treatment arms. In the better efficacy subgroup, 293/406 patients (72.2%) receiving FOLFIRI-placebo died during the observation period compared with 244/404 patients (60.4%) receiving FOLFIRI-aflibercept. There was a significant improvement in OS with aflibercept in the better efficacy subgroup (adjusted HR: 0.73; 95% CI: 0.61-0.86). The median OS was 13.1 months (95% CI: 11.7-14.2) for FOLFIRI-placebo-treated patients and 16.2 months (95% CI: 14.5-18.1) for those treated with FOLFIRI-aflibercept with an absolute difference of 3.1 months in median OS. Over time, the magnitude of survival differences between the 2 treatment groups continued to increase in the better efficacy subgroup with the absolute OS rate differences increased from 5% at 6 months to 15% at 30 months (Figure 2A). Table 3 shows a summary of efficacy results for better and poorer efficacy subgroups by treatment arms.

**Table 3:** **Summary of efficacy results for better and poorer efficacy subgroups by treatment arms**

	Better efficacy subgroup		Poor efficacy subgroup	
	FOLFIRI-placebo	FOLFIRI-aflibercept	FOLFIRI-placebo	FOLFIRI-aflibercept
Overall survival rates				
6 months	83%	88%	71%	70%
12 months	54%	64%	43%	42%
18 months	34%	46%	24%	24%
24 months	22%	35%	12%	15%
30 months	12%	27%	10%	13%
Progression free survival rates				
6 months	38%	63%		
12 months	15%	20%		
18 months	5%	6%		
Objective response rates				
	11%	24%		

**Figure 3:**
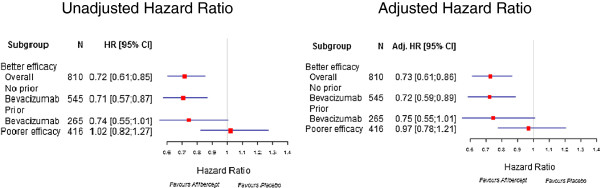
**Forest plot of overall survival for the better and poorer efficacy subgroups.** CI, confidence interval. Adjusted HR: Hazard ratio adjusted on baseline value of performance status, prior bevacizumab, age, hypertension and number of metastatic organs.

**Figure 4:**
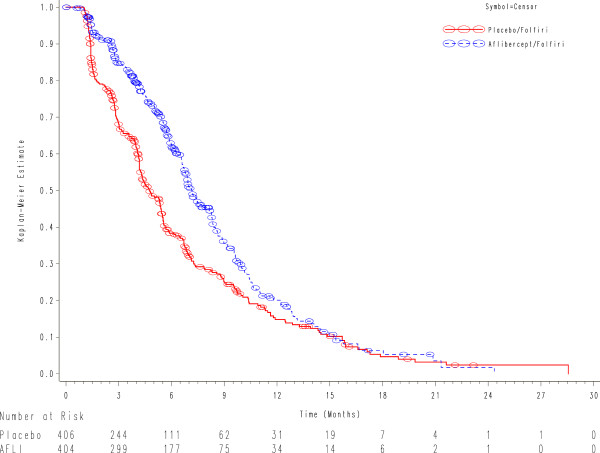
**Progression free survival for the better efficacy subgroup by treatment arms.**

Figure 2B shows the OS for the poorer efficacy subgroup by treatment arms. By contrast, in the poorer efficacy subgroup, no benefit was seen with aflibercept. 167/208 (80.3%) patients receiving FOLFORI-placebo died during the observation period versus 159/208 (76.4%) receiving FOLFIRI-aflibercept. The adjusted HR for FOLFIRI-aflibercept versus FOLFIRI-placebo was 0.97 (95% CI: 0.78-1.21). The median OS was 10.4 months (95% CI: 9.5-12.1) for FOLFIRI-placebo and 9.6 months (95% CI: 8.6-11.5) for FOLFIRI-aflibercept. There were no notable absolute survival differences between the two treatment arms in the poorer efficacy subgroup (Figure 2B and Table 3). Figure 3 shows a forest plot of overall survival for the better and poorer efficacy subgroups. Survival was similar in the better efficacy subgroup irrespective of prior bevacizumab use.In addition, for the better efficacy subgroup, a significant improvement in PFS was also observed for FOLFIRI-aflibercept. The adjusted HR for FOLFIRI-aflibercept versus FOLFIRI-placebo for PFS was 0.68 (95% CI: 0.57-0.80). Figure 4 shows the PFS for the better efficacy subgroup by treatment arms. The median PFS for FOLFIRI-placebo was 4.8 months (95% CI: 4.2-5.4) compared with 7.2 months for FOLFIRI-aflibercept (95% CI: 6.8-8.2), an absolute difference of 2.4 months in median PFS. The absolute difference in 6-month PFS rates was 25%. Similarly, for the better efficacy subgroup, the objective response rate (ORR) results followed a consistent pattern: 23.7% (95% CI: 19.3-28.2) for FOLFIRI-aflibercept and 11% for FOLFIRI-placebo (95% CI: 7.8-14.3). On the contrary, no improvements in PFS or ORR were observed with aflibercept in the poorer efficacy subgroup.

Dose intensities were similar between better and poorer efficacy groups and therefore could not account for the differential survival effect of aflibercept (Additional file 1: Table S1). However, as the better efficacy group had a longer PFS, the mean number of cycles of aflibercept was more for the better efficacy subgroup.

### Safety and tolerability

Additional file 1: Table S2 shows adverse reactions and abnormalities in laboratory values (all grades) reported at a higher incidence (≥2%) in patients treated with aflibercept plus FOLFIRI compared with placebo plus FOLFIRI in the overall safety population as well as the better and poorer efficacy subgroups. No notable differences were seen with all grades and grade 3/4 AEs for the better and poorer efficacy subgroups compared with the overall safety population as well as between the two subgroups.

In the better efficacy subgroup, the most commonly encountered AEs were gastrointestinal in the FOLFIRI-aflibercept group as was also seen in the entire study population. Diarrhoea (all grades) occurred in approximately 70% of patients in the FOLFIRI-aflibercept group and in approximately 50% of those in the FOLFIRI-placebo group. Leucopenia (all grades) occurred in 78.5% of patients in the FOLFIRI-aflibercept group and in approximately 73% of those in the FOLFIRI-placebo group. Stomatitis and hypertension were more common with FOLFIRI-aflibercept than with FOLFIRI-placebo. Severe AEs (≥grade 3) reported in the FOLFIRI-aflibercept arm included diarrhoea, stomatitis, fatigue, hypertension, and neutropenia. However, the majority of these severe treatment-associated AEs were grade 3 and included known VEGF-inhibitor class effects, such as hypertension, proteinuria, and haemorrhage, and known chemotherapy-associated AEs, such as diarrhoea, stomatitis, infection, and neutropenia.

As in the overall safety population and the better efficacy subgroup, in the poorer efficacy subgroup leucopenia was the most commonly encountered AE with FOLFIRI-aflibercept. Diarrhoea occurred in approximately 65% in both groups. Severe AEs (≥grade 3) reported in the FOLFIRI-aflibercept arm included diarrhoea, stomatitis, fatigue, hypertension, and neutropenia. The incidence of grade ≥3 treatment-emergent AEs provided were consistent between the 2 subgroups, with slightly more grade ≥3 vomiting, decreased appetite, and weight loss in the poorer responders subgroup and more grade ≥3 proteinuria in the better efficacy subgroup.

## Discussion

The VELOUR trial demonstrated a statistically significant benefit of adding aflibercept to FOLFIRI in terms of OS, PFS, and ORR when compared with placebo plus FOLFIRI in the ITT population. This post hoc survival analysis of the VELOUR study has identified a better efficacy subgroup that may have an enhanced benefit from the addition of aflibercept to FOLFIRI. Data from the post hoc better efficacy subgroup demonstrated that patients who received FOLFIRI-aflibercept had a median OS of 16.2 months (95% CI: 14.5-18.1) compared with 13.1 months (95% CI: 11.7-14.2) for FOLFIRI-placebo, a difference of 3.1 months. The adjusted HR versus FOLFIRI-placebo was 0.73 (95% CI: 0.61-0.86] as compared to 0.82 (95% CI: 0.71-0.94) in the ITT population. In the better efficacy subgroup, survival differences increased over time reaching 15% at 30 months. The results for PFS and ORR also followed a consistent pattern of improvement in the FOLFIRI-aflibercept regimen over the FOLFIRI-placebo regimen.

The efficacy outcome benefits in the better efficacy subgroup were obtained without any compromise to safety, where the AEs in the better efficacy subgroup closely mirrored those in the overall safety population. The toxicity profile is in line with expectations for the anti-VEGF plus chemotherapy class effect seen in other studies.

With regard to metastases, the primary pre-specified analysis in the ITT population showed that patients with liver-only metastases had a better treatment efficacy. In our post hoc analysis, the prognostic factor identified related to the number of metastases (≤1 metastasis) and was not limited to liver-only metastasis or specific metastasis and sufficed for any organ involvement. Data from current post-hoc analysis, however, did provide further rationale to evaluate aflibercept in patients with liver only metastases who require downsizing conversion chemotherapy.

Subgroup analyses in clinical trials are becoming increasingly important to demonstrate the consistency of the treatment effect and may provide guidance to practising physicians. Assessment of subgroups is usually confined to pre-specified analyses and *a priori* identification at the beginning of a trial. However, given the wide heterogeneity of treatment effect in relation to tumor biology and pathophysiology in oncology, well-designed post hoc analyses in clinical trials aligned with biologic plausibility should be considered an important means to explore treatment benefits in clinically relevant subpopulations.

The findings of this study are aligned with other published data in terms of the assessment of clinical determinants of survival in mCRC. Köhne *et al*. analyzed 3,825 patients with mCRC who received treatment with 5-fluorouracil in 19 prospective, randomized, controlled trials, and identified 3 prognostic risk groups based on 4 clinical parameters that included both PS and number of metastatic sites [15]. PS0 or PS1 was found to be a positive predictor of overall survival, whereas more than 1 metastatic site was a negative predictor of overall survival. Díaz *et al*. subsequently confirmed the application of the Köhne model in a retrospective study of an mCRC patient population treated with an irinotecan-based or oxaliplatin-based chemotherapy and concluded that ECOG PS and the number of metastatic sites were 2 of the key prognostic factors for predicting OS [16].

The limitations of this study were similar to other published post hoc analyses. The findings were a result of a non *a priori* analysis, and hence could not rule out selection bias and multiplicity aspects relating to a post hoc analytic exercise. Thus this post hoc survival analysis was only hypothesis-generating and the findings need confirmation in prospective trials.

## Conclusion

The better efficacy subgroup, comprising of patients within VELOUR study exclusive of adjuvant fast relapsers and had either PS0 with any number of metastatic sites or PS1 with <2 metastatic site, might derive enhanced efficacy benefits (OS, PFS, and ORR) with the combination of aflibercept and FOLFIRI compared with FOLFIRI alone. The magnitude of OS benefit in the better efficacy subgroup appeared numerically higher than that seen in the ITT population. The identification of this better efficacy subgroup profile for second-line patients with mCRC may help to guide practitioners toward targeted use of biologicals for maximal survival gains in clinically relevant patient populations.

## Electronic supplementary material

Additional file 1: Table S1: Dose intensities for better and poorer efficacy subgroups by treatment arms. **Table S2.** Adverse reactions and abnormalities in laboratory values (all grades) reported at a higher incidence (≥2%) in patients treated with aflibercept plus FOLFIRI compared with placebo plus FOLFIRI in the overall safety population as well as the better and poorer efficacy subgroups. (DOC 160 KB)

## Abbreviations

AEs: Adverse events
CI: Confidence interval
CRC: Colorectal cancer
ECOG: Eastern Cooperative Oncology Group
FOLFIRI: 5-fluorouracil-leuocovorin-irinotecan
HR: Hazard ratio
ITT: Intent-to-treat
IV: Intravenous
mCRC: metastatic colorectal cancer
ORR: Overall response rate
OS: Overall survival
PFS: Progression-free survival
PS: Performance status
VEGF: Vascular endothelial growth factor.
