# Supplementary material for: A VELOUR post hoc subset analysis: prognostic groups and treatment outcomes in patients with metastatic colorectal cancer treated with aflibercept and FOLFIRI
Source: BMC Cancer. 2014 Aug 20;14:605. doi: 10.1186/1471-2407-14-605 (PMC4149045; doi:10.1186/1471-2407-14-605)
Supplement: Supplementary file 1 — Additional file 1: Table S1: Dose intensities for better and poorer efficacy subgroups by treatment arms. Table S2. Adverse reactions and abnormalities in laboratory values (all grades) reported at a higher incidence (≥2%) in patients treated with aflibercept plus FOLFIRI compared with placebo plus FOLFIRI in the overall safety population as well as the better and poorer efficacy subgroups. (DOC 160 KB) [file 12885_2013_4787_MOESM1_ESM.doc]

Supplementary Table 1 Dose intensities for better and poorer efficacy subgroups by treatment arms

| Better efficacy subgroup | Placebo/Folfiri  (N=401) | Aflibercept/Folfiri  (N=407) |
| --- | --- | --- |
| Number of cycles received by patient |  |  |
| Sum | 4103 | 4045 |
| Mean (SD) | 10.2 (7.8) | 9.9 (7.3) |
| Median | 9.0 | 8.0 |
| Min : Max | 1 : 67 | 1 : 35 |
|  |  |  |
| 1 | 10 (2.5%) | 22 (5.4%) |
| 2 | 17 (4.2%) | 28 (6.9%) |
| 3 | 61 (15.2%) | 42 (10.3%) |
| 4 | 19 (4.7%) | 25 (6.1%) |
| 5 | 18 (4.5%) | 29 (7.1%) |
| 6 | 29 (7.2%) | 20 (4.9%) |
| 7 | 19 (4.7%) | 18 (4.4%) |
| 8 | 22 (5.5%) | 22 (5.4%) |
| 9 | 30 (7.5%) | 21 (5.2%) |
| 10 | 15 (3.7%) | 20 (4.9%) |
| 11-15 | 84 (20.9%) | 71 (17.4%) |
| 16-20 | 40 (10.0%) | 48 (11.8%) |
| 21-25 | 17 (4.2%) | 26 (6.4%) |
| >25 | 20 (5.0%) | 15 (3.7%) |
|  |  |  |
| Duration of exposure to aflibercept/placebo (weeks) |  |  |
| Number | 401 | 407 |
| Mean (SD) | 22.9 (17.3) | 23.4 (17.0) |
| Median | 19.0 | 19.0 |
| Min : Max | 2 : 135 | 2 : 85 |
|  |  |  |
| Total cumulative dose received (mg/kg) |  |  |
| Number | 401 | 407 |
| Mean (SD) | 40.73 (31.31) | 38.36 (28.69) |
| Median | 36.00 | 32.00 |
| Min : Max | 0.6 : 266.4 | 3.8 : 140.0 |
|  |  |  |
| Actual dose intensity (mg/kg/week) |  |  |
| Number | 401 | 407 |
| Mean (SD) | 1.78 (0.25) | 1.53 (0.44) |
| Median | 1.84 | 1.65 |
| Min : Max | 0.3 : 2.1 | 0.1 : 2.1 |
|  |  |  |
| Relative dose intensity |  |  |
| Number | 401 | 407 |
| Mean (SD) | 0.89 (0.13) | 0.76 (0.22) |
| Median | 0.92 | 0.82 |
| Min : Max | 0.2 : 1.0 | 0.1 : 1.1 |

| Poorer efficacy subgroup | Placebo/Folfiri  (N=204) | Aflibercept/Folfiri  (N=204) |
| --- | --- | --- |
| Number of cycles received by patient |  |  |
| Sum | 1932 | 1587 |
| Mean (SD) | 9.5 (8.2) | 7.8 (6.7) |
| Median | 7.0 | 5.0 |
| Min : Max | 1 : 45 | 1 : 31 |
|  |  |  |
| 1 | 14 (6.9%) | 21 (10.3%) |
| 2 | 15 (7.4%) | 24 (11.8%) |
| 3 | 24 (11.8%) | 28 (13.7%) |
| 4 | 12 (5.9%) | 20 (9.8%) |
| 5 | 14 (6.9%) | 14 (6.9%) |
| 6 | 16 (7.8%) | 9 (4.4%) |
| 7 | 10 (4.9%) | 10 (4.9%) |
| 8 | 12 (5.9%) | 7 (3.4%) |
| 9 | 15 (7.4%) | 8 (3.9%) |
| 10 | 6 (2.9%) | 8 (3.9%) |
| 11-15 | 28 (13.7%) | 23 (11.3%) |
| 16-20 | 17 (8.3%) | 20 (9.8%) |
| 21-25 | 11 (5.4%) | 8 (3.9%) |
| >25 | 10 (4.9%) | 4 (2.0%) |
|  |  |  |
| Duration of exposure to aflibercept/placebo (weeks) |  |  |
| Number | 204 | 204 |
| Mean (SD) | 21.0 (18.0) | 18.3 (15.5) |
| Median | 16.0 | 12.0 |
| Min : Max | 2 : 97 | 2 : 74 |
|  |  |  |
| Total cumulative dose received (mg/kg) |  |  |
| Number | 204 | 204 |
| Mean (SD) | 37.46 (32.28) | 30.36 (25.68) |
| Median | 28.00 | 20.00 |
| Min : Max | 3.9 : 181.0 | 3.9 : 108.0 |
|  |  |  |
| Actual dose intensity (mg/kg/week) |  |  |
| Number | 204 | 204 |
| Mean (SD) | 1.78 (0.24) | 1.61 (0.43) |
| Median | 1.84 | 1.72 |
| Min : Max | 0.8 : 2.1 | 0.1 : 2.1 |
|  |  |  |
| Relative dose intensity |  |  |
| Number | 204 | 204 |
| Mean (SD) | 0.89 (0.12) | 0.81 (0.21) |
| Median | 0.92 | 0.86 |
| Min : Max | 0.4 : 1.1 | 0.1 : 1.0 |

**Supplementary Table 2 Adverse reactions and abnormalities in laboratory values (all grades) reported at a higher incidence (≥2%) in patients treated with aflibercept plus FOLFIRI compared with placebo plus FOLFIRI in the overall safety population as well as the better and poorer efficacy subgroups.**

| **Primary system organ class, preferred term,  *n* (%)** | **Overall safety population** | | | | | | **Better efficacy subgroup** | | | | | | **Poorer efficacy subgroup** | | | |
| --- | --- | --- | --- | --- | --- | --- | --- | --- | --- | --- | --- | --- | --- | --- | --- | --- |
|  | **FOLFIRI-placebo**  **(*n* = 605)** | | | | **FOLFIRI-aflibercept**  **(*n* = 611)** | | **FOLFIRI-**  **placebo**  **(*n* = 401)** | | | **FOLFIRI-aflibercept**  **(*n* = 407)** | | | **FOLFIRI-placebo**  **(*n* = 204)** | | **FOLFIRI-aflibercept**  **(*n* = 204)** | |
|  | **All grades** | | **Grades ≥3** | | **All grades** | **Grades ≥3** | **All grades** | **Grades ≥3** | | **All grades** | | **Grades ≥3** | **All grades** | **Grades ≥3** | **All grades** | **Grades**  **≥3** |
| Infections and infestations | | | | | | | | | | | | | | | | |
| Urinary tract infection | 37  (6.1) | | 5  (0.8) | 56  (9.2) | | 5  (0.8) | 25  (6.2) | 3  (0.7) | 36  (8.8) | | 3  (0.7) | | 12  (5.9) | 2  (1.0) | 20  (9.8) | 2  (1.0) |
| Nasopharyngitis | 15  (2.5) | | 0 | 28  (4.6) | | 0 | 12  (3.0) | - | 23  (5.7) | | - | | 3  (1.5) | - | 5  (2.5) | - |
| Blood and lymphatic system disorders | | | | | | | | | | | | | | | | |
| Leukopenia^a^ | 432  (72.4) | | 73  (12.2) | 472  (78.3) | | 94  (15.6) | 291  (72.9) | 51  (12.8) | 317  (78.5) | | 55  (13.6) | | 141  (71.2) | 22  (11.1) | 155  (77.9) | 39  (19.6) |
| Neutropenia^a^ | 336  (56.3) | | 176  (29.5) | 409  (67.8) | | 221  (36.7) | 227  (56.9) | 119  (29.8) | 276  (68.3) | | 142  (35.1) | | 109  (55.1) | 57  (28.8) | 133  (66.8) | 79  (39.7) |
| Thrombocytopenia^a^ | 202  (33.8) | | 10  (1.7) | 286  (47.4) | | 20  (3.3) | 147  (36.8) | 8  (2.0) | 195  (48.3) | | 14  (3.5) | | 55  (27.8) | 2  (1.0) | 91  (45.7) | 6  (3.0) |
| Febrile neutropenia | 10  (1.7) | | 10  (1.7) | 26  (4.3) | | 26  (4.3) | 6  (1.5) | 6  (1.5) | 18  (4.4) | | 18  (4.4) | | 4  (2.0) | 4  (2.0) | 8  (3.9) | 8  (3.9) |
| Metabolic and nutritional disorders | | | | | | | | | | | | | | | | |
| Decreased appetite | | 144  (23.8) | 11  (1.8) | 195  (31.9) | | 21  (3.4) | 92  (22.9) | 6  (1.5) | 129  (31.7) | | 10  (2.5) | | 52  (25.5) | 5  (2.5) | 66  (32.4) | 11  (5.4) |
| Dehydration | | 18  (3.0) | 8  (1.3) | 55  (9.0) | | 26  (4.3) | 7  (1.7) | 4  (1.0) | 31  (7.6) | | 16  (3.9) | | 11  (5.4) | 4  (2.0) | 24  (11.8) | 10  (4.9) |
| Nervous system disorders | | | | | | | | | | | | | | | | |
| Headache | | 53  (8.8) | 2  (0.3) | 136  (22.3) | | 10  (1.6) | 36  (9.0) | 1  (0.2) | 97  (23.8) | | 8  (2.0) | | 17  (8.3) | 1  (0.5) | 39  (19.1) | 2  (1.0) |
| Vascular disorders | | | | | | | | | | | | | | | | |
| Hypertension | | 65  (10.7) | 9  (1.5) | 252  (41.2) | | 117  (19.1) | 43  (10.7) | 5  (1.2) | 178  (43.7) | | 80  (19.7) | | 22  (10.8) | 4  (2.0) | 74  (36.3) | 37  (18.1) |
| Respiratory, thoracic, and mediastinal disorders | | | | | | | | | | | | | | | | |
| Epistaxis | | 45  (7.4) | – | 169  (27.7) | | 1  (0.2) | 32  (8.0) | – | 125  (30.7) | | 1  (0.2) | | 13  (6.4) | – | 44  (21.6) | – |
| Dysphonia | | 20  (3.3) | 0 | 155  (25.4) | | 3  (0.5) | 11  (2.7) | - | 110  (27.0) | | 3  (0.7) | | 9  (4.4) | - | 45  (22.1) | - |
| Dyspnea | | 52  (8.6) | 5  (0.8) | 72  (11.8) | | 5  (0.8) | 28  (7.0) | 1  (0.2) | 45  (11.1) | | 3  (0.7) | | 24  (11.8) | 4  (2.0) | 27  (13.2) | 2  (1.0) |
| Oropharyngeal pain | | 19  (3.1) | - | 46  (7.5) | | 1  (0.2) | 9  (2.2) | - | 33  (8.1) | | - | | 10  (4.9) | - | 13  (6.4) | 1  (0.5) |
| Rhinorrhea | | 11  (1.8) | - | 38  (6.2) | | - | 7  (1.7) | - | 24  (5.9) | | - | | 4  (2.0) | - | 14  (6.9) | - |
| Gastrointestinal disorders | | | | | | | | | | | | | | | | |
| Diarrhea | | 342  (56.5) | 47  (7.8) | 423  (69.2) | | 118  (19.3) | 212  (52.9) | 30  (7.5) | 290  (71.3) | | 76  (18.7) | | 130  (63.7) | 17  (8.3) | 133  (65.2) | 42  (20.6) |
| Stomatitis | | 199  (32.9) | 28  (4.6) | 306  (50.1) | | 78  (12.8) | 134  (33.4) | 17  (4.2) | 209  (51.4) | | 53  (13.0) | | 65  (31.9) | 11  (5.4) | 97  (47.5) | 25  (12.3) |
| Abdominal pain | | 143  (23.6) | 14  (2.3) | 164  (26.8) | | 27  (4.4) | 95  (23.7) | 9  (2.2) | 113  (27.8) | | 13  (3.2) | | 48  (23.5) | 5  (2.5) | 51  (25.0) | 14  (6.9) |
| Abdominal pain upper | | 48  (7.9) | 6  (1.0) | 66  (10.8) | | 7  (1.1) | 33  (8.2) | 5  (1.2) | 47  (11.5) | | 2  (0.5) | | 15  (7.4) | 1  (0.5) | 19  (9.3) | 5  (2.5) |
| Hemorrhoids | | 13  (2.1) | - | 35  (5.7) | | - | 8  (2.0) | - | 27  (6.6) | | - | | 5  (2.5) | - | 8  3.9 | - |
| Rectal hemorrhage | | 15  (2.5) | 3  (0.5) | 32  (5.2) | | 4  (0.7) | 8  (2.0) | 2  (0.5) | 20  (4.9) | | 2  (0.5) | | 7  (3.4) | 1  (0.5) | 12  (5.9) | 2  (1.0) |
| Proctalgia | | 11  (1.8) | 2  (0.3) | 32  (5.2) | | 2  (0.3) | 9  (2.2) | 2  (0.5) | 23  (5.7) | | 2  (0.5) | | 2  (1.0) | - | 9  (4.4) | - |
| Aphthous stomatitis | | 14  (2.3) | - | 30  (4.9) | | 4  (0.7) | 13  (3.2) | - | 21  (5.2) | | 3  (0.7) | | 1  (0.5) | - | 9  (4.4) | 1  (0.5) |
| Toothache | | 5  (0.8) | - | 19  (3.1) | | - | 3  (0.7) | - | 15  (3.7) | | - | | 2  (1.0) | - | 4  (2.0) | - |
| Skin and subcutaneous tissue disorders | | | | | | | | | | | | | | | | |
| Palmar-plantar erythrodysesthesia syndrome | | 26  (4.3) | 3  (0.5) | 67  (11.0) | | 17  (2.8) | 17  (4.2) | 3  (0.7) | 51  (12.5) | | 14  (3.4) | | 9  (4.4) | - | 16  (7.8) | 3  (1.5) |
| Skin hyperpigmentation | | 17  (2.8) | - | 50  (8.2) | | - | 11  (2.7) | - | 39  (9.6) | | - | | 6  (2.9) | - | 11  (5.4) | - |
| Renal and urinary disorders | | | | | | | | | | | | | | | | |
| Proteinuria^b^ | | 246  (40.7) | 7  (1.2) | 380  (62.2) | | 48  (7.9) | 165  (41.1) | 7  (1.7) | 257  (63.1) | | 35  (8.6) | | 81  (39.7) | - | 123  (60.3) | 13  (6.4) |
| Serum creatinine increased^a^ | | 108  (18.1) | 3  (0.5) | 136  (22.6) | | - | 66  (16.6) | 2  (0.5) | 90  (22.3) | | - | | 42  (21.2) | 1  (0.5) | 46  (23.4) | - |
| General disorders and administration site conditions | | | | | | | | | | | | | | | | |
| Fatigue | | 236  (39.0) | 47  (7.8) | 292  (47.8) | | 77  (12.6) | 153  (38.2) | 23  (5.7) | 201  (49.4) | | 52  (12.8) | | 83  (40.7) | 24  (11.8) | 91  (44.6) | 25  (12.3) |
| Asthenia | | 80  (13.2) | 18  (3.0) | 112  (18.3) | | 31  (5.1) | 45  (11.2) | 8  (2.0) | 69  (17.0) | | 21  (5.2) | | 35  (17.2) | 10  (4.9) | 43  (21.1) | 10  (4.9) |
| Investigations | | | | | | | | | | | | | | | | |
| AST increased^a^ | | 296  (50.2) | 10  (1.7) | 339  (57.5) | | 18  (3.1) | 203  (51.8) | 8  (2.0) | 230  (57.8) | | 13  (3.3) | | 93  (47.0) | 2  (1.0) | 109  (56.8) | 5  (2.6) |
| ALT increased^a^ | | 221  (37.1) | 13  (2.2) | 284  (47.3) | | 16  (2.7) | 149  (37.5) | 12  (3.0) | 198  (49.1) | | 13  (3.2) | | 72  (36.4) | 1  (0.5) | 86  (43.7) | 3  (1.5) |
| Weight decrease | | 87  (14.4) | 5  (0.8) | 195  (31.9) | | 16  (2.6) | 54  (13.5) | 1  (0.2) | 119  (29.2) | | 8  (2.0) | | 33  (16.2) | 4  (2.0) | 76  (37.3) | 8  (3.9) |

Abbreviations: FOLFIRI, 5-fluorouracil–leucovorin–irinotecan.

^a^Based on laboratory values.

^b^Compilation of clinical and laboratory data.
